# An Improved Artificial Bee Colony Algorithm with a Probabilistic Crossover and Lock Mechanism

**DOI:** 10.3390/biomimetics11030187

**Published:** 2026-03-04

**Authors:** Zeynep Haber, Harun Uguz, Huseyin Hakli

**Affiliations:** 1 Department of Computer Engineering, Konya Technical University, 42130 Konya, Turkey; huguz@ktun.edu.tr; 2 Department of Computer Engineering, Necmettin Erbakan University, 42090 Konya, Turkey; hhakli@erbakan.edu.tr

**Keywords:** artificial bee colony, multi-resource allocation, liquid transportation, crossover operator, lock mechanism

## Abstract

The Artificial Bee Colony (ABC) algorithm is a simple and effective population-based optimization method, but it may exhibit unstable convergence and weak exploitation capability in discrete and highly constrained problems. This study proposes an improved ABC framework that integrates a probabilistic Uniform crossover operator and a gene-level lock mechanism to enhance convergence stability and local refinement. The framework is applied to an integrated multi-resource allocation problem in liquid transportation, which has not previously been addressed within the ABC literature. The problem requires the simultaneous assignment of drivers, trucks, trailers, and ISO tanks under operational and regulatory constraints. Comparative analysis of different ABC configurations shows that integrating only Uniform crossover reduced the mean cost to 17.78, adding only the lock mechanism reduced it to 29.78, and combining both further decreased it to 14.94, indicating a complementary effect between the two mechanisms. The proposed configuration consistently achieved the lowest mean costs across small, medium, and large datasets. Compared with established metaheuristic algorithms and expert manual planning (34.72), the method produced lower-cost and feasible solutions, demonstrating both algorithmic robustness and practical relevance.

## 1. Introduction

Metaheuristic algorithms have become an important research area in recent years due to their successful results in solving many problems [1]. In cases where traditional optimization approaches are limited, especially in nonlinear, high-dimensional, and large-scale problems, these nature-inspired methods have provided flexible and powerful search mechanisms [2,3,4].

One of the best examples of problems that traditional methods have difficulty solving is integer programming problems. Due to their NP-hard nature, classical methods such as dynamic programming and branch-and-bound are effective for small-scale problems, but become insufficient as problem size increases because computation time grows significantly [5,6,7]. Metaheuristic algorithms stand out at this point by producing high-quality solutions within an acceptable time. Due to these features, metaheuristic methods have attracted significant attention in the literature. Today, more than 500 metaheuristic algorithms have been proposed and examined across various application areas [8]. An important class of metaheuristic methods is composed of swarm intelligence-based algorithms [9]. These algorithms ensure diversity and adaptability in the search process by leveraging principles of cooperation and collective learning; thus, they effectively maintain a balance between exploration and exploitation. Since the 2000s, many new methods have been proposed within this group [10]. Among swarm-based approaches, widely studied algorithms such as Particle Swarm Optimization (PSO) [11], Bacterial Foraging Optimization (BFO) [12], Artificial Bee Colony (ABC) [13], Firefly Algorithm (FA) [14] and Bat Algorithm (BAT) [15] have demonstrated strong performance across various optimization problems.

Among these methods, the ABC algorithm proposed by Karaboga [13] stands out among swarm-based approaches in terms of the number of publications. It shows that researchers have confidence in the algorithm and that it has been successfully applied to various problems. The simple structure of ABC, its low parameter requirements, robustness, and strong exploration capability have made it one of the prominent methods in the literature [5,16]. Over the past two decades, ABC and its variants have been applied in a wide range of fields, including image processing [17,18,19], power systems [20,21], clustering [22,23], cybersecurity [24,25,26], vehicle routing and scheduling [27,28], revealing the versatile structure of the algorithm.

Although the ABC algorithm inherently possesses strong exploration capability, it faces issues such as instability in convergence speed and premature stagnation due to its limited exploitation ability [5,29]. Since a new food source in ABC is generated randomly, it is not clear in which direction the algorithm moves toward better solutions. This causes instability in the search process [29]. As a result, the direction selection based on randomness cannot guarantee that the solutions will always concentrate in potentially good regions [30]. Numerous improvements have been proposed in the literature to overcome these weaknesses. Among them, the hybridization of the ABC algorithm with other heuristic methods has been one of the most common approaches. For example, Zhu and Kwong [31] integrated the global best solution information of PSO into ABC; Gao and Liu [32] proposed a differential evolution-based update; Roeva et al. [33] developed a hybrid structure with a genetic algorithm; and Yildizdan and Baykan [34] introduced a hybrid model with the Bat Algorithm (BA). In addition, studies focusing on new search strategies and neighborhood structures have also aimed to improve the performance of the algorithm. For instance, Zeng et al. [35] proposed a dynamic search strategy by monitoring success rates; Li et al. [36] enhanced exploitation by combining “superior components” from successful solutions; and Lu et al. [37] proposed variants that accelerated convergence by modifying the update equations.

Recent studies have also proposed learning-enhanced ABC variants. For example, a dendrite net learning multi-objective ABC was introduced for UAV path planning, integrating a dendrite logic network to guide the search process and improve multi-objective performance [38]. These studies reflect ongoing efforts to strengthen ABC through hybrid and adaptive mechanisms.

ABC was originally developed for continuous-parameter optimization problems, and certain adaptations are required for its application to discrete problems [39]. In this regard, integer encoding, permutation-based representations, and special neighborhood structures have been developed in the literature, and various ABC variants have been applied to problems such as the Traveling Salesman Problem (TSP), job scheduling, Vehicle Routing Problem (VRP), and Capacity Allocation Problem (CAP) [10].

Existing studies on multi-resource allocation primarily focus on two-resource combinations such as driver–vehicle or vehicle–route matching [40,41,42,43,44,45,46]. However, to the best of our knowledge, integrated allocation models that simultaneously consider driver–truck–trailer–ISO tank compatibility under regulatory constraints have not been reported in the existing literature.

In this study, we investigate a real-world problem involving multi-resource assignment in liquid transportation, which, to the best of our knowledge, has not been previously examined in the literature. The problem has a complex and discrete structure that involves the simultaneous allocation of drivers, trucks, trailers, and ISO tanks. In this context, a new approach for adapting the ABC algorithm to integer problems has been proposed. In the proposed method, the crossover and lock mechanisms have been integrated to enhance ABC’s exploration and exploitation capabilities. The crossover operator combines gene segments from different candidate solutions to generate new individuals, thereby strengthening diversity in the solution space. This produces less destructive and more structure-consistent movements compared to classical difference-based updates. The lock mechanism prevents the modification of deteriorating genes for a certain number of iterations, ensuring stability in the search process. The proposed method has been applied to the multi-resource assignment problem in liquid transportation, and under real operational and regulatory constraints, it has produced higher-quality and more stable solutions for the simultaneous allocation of drivers, vehicles, trailers, and ISO tanks.

The contributions of this study to the literature can be summarized as follows:

- Multi-Resource Allocation: This study models driver, truck, trailer, and ISO tank assignment within a single optimization framework. All compatibility and ADR constraints are handled simultaneously. This unified formulation reflects the operational structure of liquid transportation and differs from partial or stage-based allocation approaches.
- Methodological Innovation: In the proposed model, a crossover operator has been integrated into the ABC algorithm to overcome the limitations of the standard difference-based update. In this way, new individuals have been generated in a less destructive manner, and diversity in the solution space has been maintained.
- Search Stability: Through the lock mechanism added to the algorithm, the modification of deteriorating genes for a certain number of iterations has been prevented, making the search process more stable and reliable. This mechanism has delayed premature convergence and enabled a more in-depth exploration.
- Application and Constraints: This study has been tested on a logistic scale, taking into account real operational and legal constraints. Sector-specific factors such as ADR (European Agreement concerning the International Carriage of Dangerous Goods by Road) certification requirements, equipment compatibility conditions, and capacity limitations have been integrated into the model, resulting in practically applicable outcomes.
- Performance and Contribution: The proposed approach has produced higher-quality and more balanced solutions compared to manual planning, demonstrating the applicability of ABC to multi-resource allocation problems. Thus, a scalable decision support tool has been provided for the logistics sector, and an original contribution has been made to the literature regarding the effectiveness of ABC in discrete and integer-based problems.

The rest of this paper is structured as follows. Section 2 reviews the related literature under three main themes: (i) variants and hybrid versions of the ABC algorithm, (ii) studies that adapt the ABC algorithm to discrete and integer optimization problems, and (iii) metaheuristic-based approaches for resource allocation and logistics optimization. Section 3 presents the definition and mathematical formulation of the multi-resource allocation problem investigated in this study. Section 4 describes the proposed method in detail, including the improved ABC algorithm, the crossover and lock mechanisms, and the repair strategy. Section 5 reports the experimental setup, datasets, parameter analyses, and comparative performance results. Finally, Section 6 concludes the study and offers some insights for future research.

## 2. Related Work

### 2.1. ABC and Its Variants

In recent years, many hybrid and improved approaches have been proposed to enhance the performance of the ABC algorithm. Ustun et al. [47] developed the mABC algorithm by integrating the mutation and crossover operators of the Differential Evolution (DE) algorithm into the onlooker bee phase of ABC, demonstrating that this method achieved higher accuracy and faster convergence compared to existing ABC variants on classical and CEC 2014 benchmark functions. Aslan and Karaboga [48] proposed the gdatABC method based on EEG signal decomposition for big data optimization problems, showing that this approach exhibited superior or at least competitive performance compared to original ABC variants and other metaheuristic algorithms without requiring local search methods or complex parameter tuning. Yildizhan ve Baykan [34] introduced the BA_ABC method by combining the Bat Algorithm (BA) with ABC, showing that this hybrid approach overcame BA’s weaknesses in global search and premature convergence and achieved superior results in both small- and large-scale CEC test functions as well as engineering design problems. Roeva et al. [33] developed the ABC–GA algorithm by combining ABC and GA, demonstrating that the proposed method produced faster and higher-quality solutions than competing hybrid algorithms in both benchmark functions and the parameter identification problem. Xiao et al. [49] proposed the ABCNG method, which incorporates adaptive neighborhood search and Gaussian perturbation, thereby enhancing the exploitation ability of the algorithm and avoiding stagnation while achieving competitive results against six different ABC variants. Finally, Etminaniesfahani et al. [50] proposed the ABFIA algorithm by combining the strong exploration capability of ABC with the fast convergence feature of the Fibonacci Indicator Algorithm (FIA), reporting that this hybrid approach outperformed both ABC and FIA on the CEC2019 functions and showed competitive performance with recent metaheuristic algorithms. Kiran et al. [51] proposed a multi-strategy ABC that uses several update rules with adaptive counters. The counters increase when a rule produces a better solution, helping the algorithm learn which rule works best. Tests on benchmark functions showed faster convergence and higher accuracy than standard ABC.

### 2.2. Applications of ABC for Discrete/Integer Problems

Although the ABC algorithm was originally designed for continuous optimization, numerous studies have adapted and extended it for discrete and integer programming problems. Brajević [5] proposed the SB-ABC method enhanced with a Shuffle Mutation Operator to overcome the slow convergence and weak exploitation issues of the ABC algorithm and demonstrated that this algorithm achieved superior results in both integer programming and minimax problems. Liu et al. [52] developed a new network-based MILP model and a discrete artificial bee colony (DABC) algorithm adapted for process planning and scheduling in distributed manufacturing environments, reporting that this approach outperformed strong existing methods in their tests. Similarly, Zou et al. [53] developed an MILP model and a DABC algorithm for the multi-AGV dispatching problem in matrix manufacturing workshops and achieved better results than existing methods on 110 real factory instances. Durgut and Aydin [54] proposed an adaptive hybrid ABC algorithm for binary problems, where different operator selection schemes (PM, AP, UCB) were comparatively analyzed, and better results were obtained in uncapacitated facility location problems. Alaidi et al. [39] adapted the ABC algorithm to discrete problems and developed six neighborhood operators and a crossover-based operator for the Traveling Salesman Problem (TSP), reporting promising results on TSPLIB benchmarks. Rahimi [55] also combined the ABC algorithm with DNNA for TSP, introducing 2-opt neighborhood search and new exploration strategies that provided higher-quality solutions than the basic ABC and improved the best-known results for some problems. Finally, Li et al. [56] addressed the distributed heterogeneous no-wait flowshop scheduling problem by developing a new MILP model and a DABC algorithm enhanced with four different neighborhood searches, showing that higher-quality results than the best-known solutions were obtained in both small- and large-scale tests.

### 2.3. Logistics and Resource Allocation Optimization with Metaheuristics

Liquid transportation operations are not limited to the planning of a single resource but require the simultaneous and coordinated management of multiple elements, such as drivers, vehicles, trailers, and auxiliary equipment. Although studies on multi-source planning in liquid transportation are limited in the literature, various studies have been conducted in different sectors.

Torrance et al. [40] addressed driver and vehicle scheduling in the context of logistics and public transportation systems, focusing on the optimization of shift durations, rest breaks, legal restrictions, routes, trip frequencies, and cycle times. Melemez et al. [41] developed an ergonomic and cost-effective solution for log trailer design in Turkey’s forest harvesting operations by integrating QFD, TRIZ, and AHP methods. Cavone et al. [57] modeled freight transfer operations in intermodal terminals using Timed Petri Nets and analyzed performance bottlenecks and additional resource requirements through Monte Carlo simulations. Bula et al. [58] determined the distribution method for cement blocks using the AHP method and presented a practical decision support approach for small and medium-sized enterprises. Evaluated criteria such as tare weight, capacity, service network, and fleet compatibility in the selection of heavy trailers using the AHP method. Aliano et al. [43] also assessed heavy trailer selection based on criteria similar to those of the AHP method. Gorcun et al. [44] proposed a reliable model for hazardous material transportation by combining fuzzy SWARA and CODAS methods to account for uncertainty in tanker selection. Feng and Cheng [45] developed a two-stage model and a genetic algorithm-based solution for truck–load matching in drop-and-pull transportation. Lee et al. [59] improved economic and operational efficiency in industrial gas supply chains with a MINLP model integrating production, distribution, and transport resource allocation. Makhova et al. [46] developed cost-, time-, and quality-oriented optimization models for truck–load matching in Russia and evaluated fleet utilization scenarios using GA-based simulations. Finally, Maroof et al. [60] proposed a hybrid GA–Solomon approach for the vehicle routing problem with time windows, achieving Best Known Solutions in benchmark tests and demonstrating competitiveness with advanced techniques in the literature.

In this context, the literature includes various studies on resource planning in logistics, often developed within specific sectors or focused on particular resource types. Research addressing fully integrated coordination structures in liquid transportation remains relatively limited. The present study introduces a comprehensive planning model in which drivers, trucks, trailers, and ISO tanks are considered together within the same decision framework.

Several logistics allocation studies examine limited resource combinations such as driver–vehicle or vehicle–route matching. In some cases, allocation decisions are structured in stages, where different resource types are determined separately. While such approaches provide practical modeling advantages, they may not fully reflect the operational interdependencies observed in liquid transportation systems.

In liquid transportation planning, driver, truck, trailer, and ISO tank decisions are closely connected. Regulatory requirements such as ADR certification restrict feasible assignments. Compatibility conditions, location constraints, and workload balance must be evaluated simultaneously. The present study formulates these elements within a single integrated optimization model. All resource types are assigned concurrently, and operational as well as regulatory constraints are evaluated within one unified structure. This modeling perspective differentiates the proposed framework from allocation approaches that treat resources separately or in partially coordinated settings.

### 2.4. Research Gap and Positioning of the Proposed Study

The existing literature reveals two main research streams relevant to this study. The first focuses on improving the ABC algorithm through adaptive parameter control, hybridization with other metaheuristics, or operator redesign for discrete problems. The second stream addresses resource allocation and logistics planning problems, typically concentrating on limited resource combinations such as driver–vehicle or vehicle–route matching.

From an algorithmic perspective, many ABC variants focus on adaptive parameter control, dynamic rule selection, or hybridization with other metaheuristics such as PSO, DE, and GA. Adaptive approaches typically modify control parameters during iterations, while hybrid models embed external search operators into the ABC framework. Discrete adaptations, on the other hand, often redesign neighborhood operators to handle combinatorial decision variables.

However, limited attention has been given to the structural mechanisms that regulate gene-level modification behavior while preserving the original ABC cycle. In particular, simple and operator-independent mechanisms that temporarily prevent repeated unsuccessful updates, without introducing adaptive parameter schemes, external hybridization, or tabu-style historical memory structures, remain largely unexplored. The proposed probabilistic crossover and gene-level lock strategy address this gap by directly controlling variable updates and improving convergence behavior in discrete search spaces, while maintaining the core employee–onlooker–scout structure of ABC.

From a problem perspective, most studies in logistics optimization consider partial resource combinations, such as driver–vehicle matching or vehicle–route assignment. Integrated models that simultaneously coordinate drivers, trucks, trailers, and ISO tanks under operational and regulatory (ADR) constraints are rarely addressed, particularly within the ABC literature.

This study contributes to both dimensions. Algorithmically, it introduces a structured gene-level control mechanism that reshapes the exploration–exploitation balance without altering the fundamental ABC workflow. Problem-wise, it presents, to the best of our knowledge, the first ABC-based framework for integrated multi-resource assignment in liquid transportation, incorporating real operational and regulatory constraints.

## 3. Problem Formulation

### 3.1. Problem Definition

Liquid transportation deals with the safe and efficient transfer of fluid materials. Within this scope, petroleum derivatives, various chemicals, liquid food products, and gaseous substances are transported and shipped through different modes of transportation, including road, sea, rail, and air.

In road transportation, the shipment of these products is generally carried out through the coordinated use of equipment such as trucks, trailers, and ISO tanks. All operational data used in this study were obtained from a large-scale logistics company operating in Turkey; thus, the planning challenges, constraints, and operational processes are directly based on real field experiences. Each resource in the system has distinct characteristics. For example, drivers are responsible not only for transporting the cargo but also for loading and unloading operations; therefore, they must receive specialized training. ISO tanks are equipment used for liquid and gas transportation, manufactured in accordance with international standards to withstand high pressure and hazardous contents. Trailers are carrier units that transport the tanks and may offer either standard or tanker-type integrated configurations. Trucks that tow these units must be ADR-certified, as this certification is mandatory for hazardous material transport.

In the selection of each resource type, capacity information and technical, legal, and operational criteria are taken into account. For trucks, brand, model, ADR compliance, and mileage are important; for trailers, the associated tank number and ADR compliance are determining factors. For ISO tanks, cleaning records, the last transported product, volume, and facility location are of critical importance. For drivers, license types, ADR qualifications, and performance evaluations are considered. Summary information regarding the main resources used within this scope is presented in Table 1.

In addition to resource characteristics, customer orders are also a key factor in the planning process. Orders include information such as the product to be transported, quantity, loading and delivery dates and locations, ADR compliance, and priority level. This information is crucial for assigning the correct resource combinations and ensuring timely delivery.

**Figure 1:**
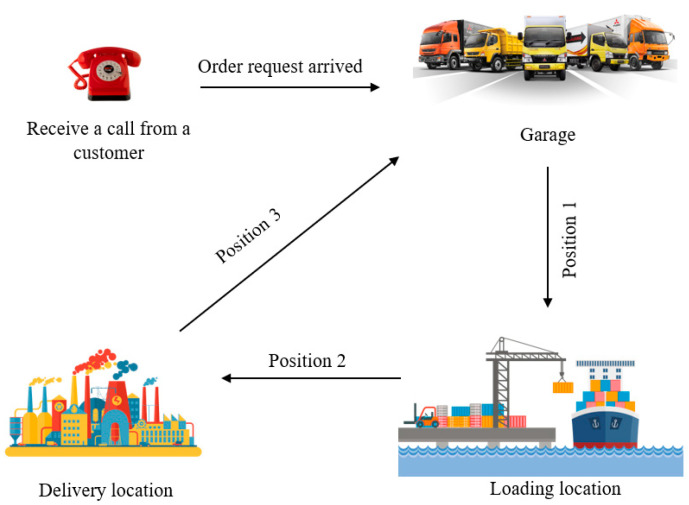
Order–Loading–Delivery Flow Cycle in Liquid Transportation Operations.

The typical flow of the shipment process is summarized in Figure 1. The process begins when the order is received by the logistics company and continues with the identification of suitable vehicles and equipment in the garage, which are then directed to the loading area (Position 1). After loading is completed, the product is transported to the customer location (Position 2), and upon delivery, the vehicles return to the garage to be prepared for new orders (Position 3).

The resource-allocation problem for liquid transportation is a highly constrained optimization problem that requires assigning various resources, including drivers, trucks, trailers, and ISO tanks, to incoming orders within specific time periods. The dependencies among these resources lead to an exponential increase in feasible combinations, making the problem highly complex. In this problem, operational feasibility, the minimization of costs, efficient utilization of resources, and compliance with legal and safety constraints must be ensured simultaneously.

### 3.2. Mathematical Model

The simultaneous assignment of driver–truck–trailer–ISO tank resources and the maintenance of load balance in liquid transportation are represented by the mathematical formulation presented below. First, the indices, sets, and parameters are defined, and then the functions describing order–resource matching are introduced. Subsequently, eight objective functions are introduced, jointly considering fuel consumption, distance, position distributions, and intra-city ratio targets based on historical operational data.

#### 3.2.1. Constraints

The mathematical model, a set of constraints is defined to ensure the valid allocation of drivers, trucks, trailers, and ISO tanks to orders, and Table 2 reports these constraints together with their mathematical formulations and interpretations.

**Table 2:** Constraints of the Problem.

$\sum_{j,v,k,t,s}x_{i,j,v,k,t,s}=1,\;\forall i$	(1)
$\sum_{i,v,k,t}x_{i,j,v,k,t,s}\leq 1,\;\forall u,s$	(2)
$\sum_{i,j,k,t}x_{i,j,v,k,t,s}\leq 1,\;\forall v,s$	(3)
$\sum_{i,j,v,k}x_{i,j,v,k,t,s}\leq 1,\;\forall t,s$	(4)
$\sum_{i,j,v,t}x_{i,j,v,k,t,s}\leq 1,\;\forall k,s$	(5)
$x_{i,j,v,k,t,s}=0\;,\;if\;city\left(v\right)\neq city(t)$ $x_{i,j,v,k,t,s}=0,\;\mathrm{if}\;\left[city\left(v\right)\neq city\left(k\right)\right]\;\wedge \;[city(i)\neq city(k)]$	(6)
$x_{i,j,v,k,t,s}=0,\forall i\in N_{ADR},\;t\in T_{noADR},\;j,v,k,s$	(7)

The constraints presented in Table 2 ensure the feasibility and consistency of the assignment decisions. Constraint (1) guarantees that each order is assigned to exactly one feasible combination of driver, truck, trailer, and ISO tank within the planning period. Constraints (2)–(5) enforce resource exclusivity by ensuring that each driver, truck, trailer, and ISO tank can be allocated to at most one order during the same time period. Constraint (6) establishes location compatibility conditions, requiring the truck and trailer to be in the same city and ensuring that the ISO tank is appropriately located relative to the order’s loading point. Finally, Constraint (7) enforces regulatory compliance by preventing orders requiring ADR certification from being assigned to vehicles that lack the necessary ADR documentation. Together, these constraints maintain operational consistency, resource feasibility, and regulatory compliance within the proposed optimization framework.

#### 3.2.2. Objective Functions

The simultaneous assignment of driver–truck–trailer–ISO tank resources and the maintenance of load balance in liquid transportation are defined by the eight sub-objectives presented below. Each sub-objective represents a different dimension of operational balance.

(8) $$Z=\min\left(\sum_{k=1}^{8}z_{k}\right)$$

The objective function aims to minimize the total cost, which consists of eight components. The components, denoted as z_1_–z_8_, are defined below and have all been normalized; therefore, their simple summation is used.

Although the problem involves multiple performance criteria, a weighted-sum formulation was adopted instead of a Pareto-based multi-objective framework. In practical logistics planning, decision-makers typically require a single implementable solution rather than a set of Pareto-optimal alternatives. The weighted-sum structure allows the integration of operational priorities directly into the objective function through explicit weight parameters assigned to each normalized objective, thereby reflecting managerial preferences. Moreover, this approach simplifies the decision process and avoids the need to maintain and evaluate a population of non-dominated solutions, as required in true multi-objective evolutionary algorithms. Nevertheless, extending the proposed framework to a Pareto-based multi-objective ABC variant constitutes a promising direction for future research.

Objective 1: Fuel/Distance Normalization

This objective evaluates fuel efficiency in truck/order matching. The relationship between the total fuel consumption of the trucks assigned to incoming orders and the total distance of those orders is normalized by using the truck with the highest fuel consumption as the reference. A lower value of $z_{1}$ indicates more efficient system operation.

(9) $${y_{max}=max}_{v=1,\ldots ,m_{c}}\{y_{v}\}$$

(10) $$F=\sum_{j=1}^{n}d_{i}y_{\tau (j)}$$

(11) $$D_{tot}=\sum_{j=1}^{n}d_{j}$$

(12) $$z_{1}=\frac{F}{y_{max}D_{tot}}$$

Objective 2: Driver Distance Balance

The total driving distance of each driver is minimized so that it remains close to the average distance.

(13) $$K_{d}^{past}=\sum_{u=1}^{m_{d}}K_{u}^{past}$$

(14) $$\mu_{d}=\frac{K_{d}^{past}+D_{tot}}{m_{d}}$$

(15) $$M_{i}=K_{u}^{past}+\sum_{j:\delta \left(j\right)=u}d_{j}$$

(16) $$z_{2}=\frac{\sum_{i=1}^{k}\left|\mu_{d}-M_{i}\right|}{\mu_{d}}$$

Objective 3: Short/Long Trip Ratio Balance

This objective aims to balance the distribution of short and long trips for each driver. The ratio of short-to-long trips for each driver is balanced around the target value of 0.5. Thus, as $z_{3}$ decreases, short and long trips are distributed more fairly and evenly among drivers.

(17) $$S_{u}^{new}=\sum_{j:\delta \left(j\right)=u}X_{j}^{short}$$

(18) $$N_{u}^{new}=\left|\left\{j:\delta \left(j\right)=u\right\}\right|$$

(19) $$p_{u}=\frac{S_{u}^{new}+S_{u}^{past}}{S_{u}^{past}+L_{u}^{past}+N_{u}^{new}}$$

(20) $$z_{3}=\sum_{u=1}^{m_{d}}\left|0.5-p_{u}\right|$$

Objective 4: Trailer Position Balance

The number of positions assigned to each trailer is balanced to be close to the average for all trailers. This equalizes the workload between trailers, preventing any trailer from being over- or under-positioned relative to the others.

(21) $$P_{tot}^{past}=\sum_{t=1}^{m_{t}}P_{t}^{past}$$

(22) $$Q_{t}^{new}=3n$$

(23) $$\mu_{t}=\frac{P_{tot}^{past}+Q_{t}^{new}}{m_{t}}$$

(24) $$P_{t}=P_{t}^{past}+3\sum_{j:\theta \left(j\right)=t}1$$

(25) $$z_{4}=\frac{\sum_{t=1}^{m_{t}}\left|\mu_{t}-P_{t}\right|}{\mu_{t}}$$

Objective 5: Trailer Intra-City Ratio Target

The intra-city utilization ratio of each trailer is balanced to meet the target ratio φ. The parameter φ represents the desired proportion of intra-city assignments relative to total trailer operations. In this study, φ was fixed at 0.4 based on historical operational data provided by the collaborating logistics company. This value reflects the company’s long-term average intra-city utilization benchmark, and deviations from this target are penalized in the objective function to maintain operational balance.

(26) $$C_{t}=P_{t}^{past,city}+3\sum_{j:\theta \left(j\right)=t}X_{j}^{city}$$

(27) $$R_{t}=\frac{C_{t}}{P_{t}}$$

(28) $$z_{5}=\sum_{t=1}^{\mu_{t}}max\{0,\;\varphi -R_{t}\}\;$$

Objective 6: Truck Distance Balance

The total driving distance of each truck is minimized so that it does not deviate from the average distance.

(29) $$K_{c}^{past}=\sum_{v=1}^{m_{c}}K_{v}^{past}$$

(30) $$\mu_{c}=\frac{K_{c}^{past}+D_{tot}}{m_{c}}$$

(31) $$K_{v}=K_{v}^{past}+\sum_{j:\tau \left(j\right)=v}d_{j}$$

(32) $$z_{6}=\frac{\sum_{v=1}^{m_{c}}\left|\mu_{c}-K_{v}\right|}{\mu_{c}}$$

Objective 7: ISO Tank Position Balance

The number of positions assigned to each ISO tank is balanced so that it remains close to the average value.

(33) $$Q_{tot}^{past}=\sum_{k=1}^{m_{i}}Q_{k}^{past}$$

(34) $$Q_{i}^{new}=3n$$

(35) $$\mu_{i}=\frac{Q_{tot}^{past}+Q_{i}^{new}}{m_{i}}$$

(36) $$Q_{k}=Q_{k}^{past}+3\sum_{j:\kappa \left(j\right)=k}1$$

(37) $$z_{7}=\frac{\sum_{i=1}^{m_{i}}\left|\mu_{i}-Q_{k}\right|}{\mu_{i}}$$

Objective 8: ISO Tank Intra-City Ratio Target

The intra-city utilization ratio of each ISO tank is balanced to meet the target ratio φ.

(38) $$C_{k}=Q_{k}^{past,city}+3\sum_{j:\kappa \left(j\right)=k}X_{j}^{city}$$

(39) $$R_{k}=\frac{C_{k}}{Q_{k}}$$

(40) $$z_{8}=\sum_{k=1}^{m_{i}}max\{0,\;\varphi -R_{k}\}$$

The eight defined objectives collectively aim to ensure both operational efficiency and balanced utilization of all resources.

## 4. Methodology

### 4.1. Original ABC

The ABC was proposed by Karaboga in 2005 [13]. It is a swarm-based optimization method inspired by the behavior of honeybees. Bees are divided into three roles: employee, scout, and onlooker to discover and refine candidate solutions.

In the ABC initialization phase, a certain number of random candidate solutions are generated, and their fitness is calculated. In the employee bee phase, a new candidate solution is generated for each solution using Equation (41). If the new candidate solution produces a better result, an update is performed; otherwise, the solution’s trial counter is incremented. In the onlooker bee phase, solutions are selected with probabilities proportional to their fitness, and the search for the solution space continues with similar neighborhood searches. The scout bee phase, on the other hand, restores population diversity by completely recreating solutions that have not been improved for a long time. This triple mechanism naturally establishes the balance of exploration and exploitation within a simple structure:

(41) $$x_{i}^{\;new}=x_{i}+\phi (x_{i}-x_{k}),\phi \in [-1,\;1]$$

The pseudocode of the original ABC algorithm is shown in Algorithm 1.
**Algorithm 1.** Pseudocode of the original ABC algorithm1:   Initialize parameters (SN, limit, MaxCycle)2:   Generate initial population xi (i = 1, …, SN)3:   Evaluate Fitness (xi); trial (i) ← 04:   Best ← best solution5:   **for** cycle = 1 to MaxCycle **do**6:       // Employee phase7:       **for each** xi **do**8:        Select k ≠ i randomly9:        Generate vi by:10:                 vij = xij + φij (xij − xkj)11:        **if** Fitness (vi) ≤ Fitness (xi) **then**12:                 xi ← vi; trial (i) ← 013:        **else**14:                 trial (i) ← trial (i) + 115:        **end if**16:       **end for**17:       // Onlooker phase18:       Compute selection probabilities pi19:       **for** t = 1 to SN **do**20:        Select i using roulette(pi)21:        Select k ≠ i randomly22:        Generate vi as above23:        **if** Fitness (vi) ≤ Fitness (xi) **then**24:                 xi ← vi; trial (i) ← 025:        **else**26:                 trial (i) ← trial (i) + 127:        **end if**28:       **end for**29:       // Scout phase30:       **for each** xi with trial (i) ≥ limit **do**31:        xi ← RandomSolution (bounds); trial (i) ← 032:       **end for**33:       Update Best34:   **end for**35:   **return** Best

### 4.2. Representation and Initialization

In this study, each solution generated by the ABC algorithm is encoded as a one-dimensional integer vector. Each element of the vector represents the resource assigned to a specific order, and the resources are organized in the following order: Truck (V), Trailer (T), ISO Tank (I), and Driver (D). In this way, all order–resource assignments are combined into a single sequence and incorporated into the optimization process.

**Figure 2:**
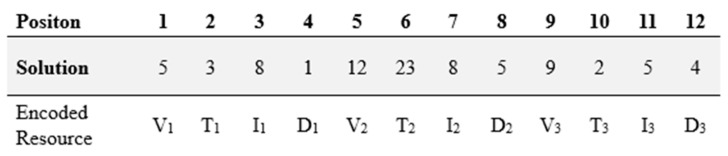
Example of Solution Encoding Representing Truck, Trailer, ISO Tank, and Driver Assignments.

Figure 2 illustrates how three orders are simultaneously assigned to four types of resources in an integer-based structure:

In Figure 2, $V_{i}$ $V_{i},T_{i},I_{i},D_{i}$ respectively represent the truck, trailer, ISO tank, and driver assigned to the $i^{th}$ order. The first order is assigned to truck 5, trailer 3, ISO tank 8, and driver 1. The second order is assigned to a combination consisting of truck 12, trailer 23, ISO tank 8, and driver 5. The third order is associated with truck 9, trailer 2, ISO tank 6, and driver 4.

Each component can take values only within the range of assignable resources. Accordingly, the lower limit (LB) is 1 for all genes, while the upper limit (UB) corresponds to the total number of resources of that type. For example, if there are 10 trailers in the dataset, the trailer genes in an individual take random values within the range of 1–10. Similarly, the genes representing drivers, trucks, and ISO tanks are defined within bounds proportional to their respective resource counts.

This representation stores all resource assignments in a single one-dimensional structure, allowing the algorithm to efficiently process individuals without the need to construct multi-dimensional data structures. During fitness evaluation, the vector is decoded, and the resource combination corresponding to each order is reconstructed. Then, the individual’s overall fitness value is calculated based on performance metrics such as fuel consumption, distance balance, resource utilization ratio, and city route ratio targets.

### 4.3. Repair and Penalty Mechanism

In order to handle constraint violations effectively, the algorithm employs a repair and penalty mechanism. If the constraints are violated, the function applies a predefined penalty score, and each violation requires a sufficiently high penalty cost ($P_{Cost}$) to discourage infeasible solutions.

In this study, $P_{Cost}$ was intentionally set to a sufficiently large value to strictly enforce feasibility. Since the objective components are normalized and remain within a limited numerical range, the penalty coefficient was selected to be significantly larger than the maximum possible aggregated objective value. Therefore, any infeasible solution is decisively dominated by feasible ones. This design reflects the operational nature of the problem, where violations such as ADR incompatibility or resource conflicts correspond to practically unacceptable solutions. Consequently, $P_{Cost}$ functions as a feasibility barrier rather than a fine-tuning parameter, and the algorithm operates primarily within the feasible region after the early search stages.

(42) $$PenaltySum=\sum_{i=1}^{n}{penalty}_{i}$$

(43) $${penalty}_{i}=\;\;\;\left\{\begin{array}{c} P_{Cost}\\ 0 \end{array}\begin{array}{c} \;\;\;\;\;\;\mathrm{If}\mathrm{at}\mathrm{least}\mathrm{one}\mathrm{constraint}\mathrm{is}\mathrm{violated}\;\\ \mathrm{Otherwise} \end{array}\;\;\right.$$

In the proposed ABC-based model, the repair mechanism ensures that each solution remains valid under the defined constraints. Throughout the search process, it continuously checks whether multiple orders overlap within the same time period for a given resource (driver, truck, trailer, or ISO tank). If the same resource is assigned to more than one order within the same period, this situation is considered a conflict, and the repair mechanism is triggered.

The repair mechanism randomly selects one of the conflicting orders and assigns it to another suitable resource. The operation of this mechanism can be illustrated using a matrix that represents order–time conflicts, as shown in Table 3.

For example, in a case with three orders, the diagonal “1” values in the matrix represent the orders themselves, while the “1” values in other cells indicate orders that overlap within the same time period. If Order 1 and Order 3 are scheduled during the same period and both are assigned to the same truck, the algorithm detects this conflict and reassigns Order 3 to a different truck. This process can be summarized by the following logic:

$$\mathrm{IF}\;overlap(order_{i},order_{j})=1\;\mathrm{and}\;sameResource(i,j)=1\Rightarrow reassign(order_{j})$$

### 4.4. Fitness

In this study, the fitness evaluation in both the original and improved ABC algorithms is based on the total cost function Z defined in Section 3.2. The fitness of each candidate solution is calculated using the normalized sum of the eight sub-objectives (z_1_–z_8_) defined in Equation (8). This structure simultaneously evaluates performance measures such as fuel consumption, distance balance, resource utilization rate, and intra-city quota targets.

The total fitness cost is calculated as the sum of the objective function value and the penalty terms resulting from constraint violations:

(44) Fitness=Z+PenaltySum

A lower fitness value indicates that the solution is more aligned with the system objectives; however, if a solution violates defined constraints (such as truck–trailer geographic compatibility or ADR compliance), a penalty coefficient (P_Cost_) is added to the model, thereby reducing the fitness of that individual. As a result, the fitness function simultaneously considers both the optimization of objective functions and the preservation of feasibility conditions.

## 5. Proposed Method

### 5.1. Improved ABC

The improved ABC method aims to address the weaknesses of the original ABC method in local exploitation, convergence speed, and applicability to integer problems. The proposed approach (i) effectively preserves both exploration and local optimization by establishing a dynamic balance with probabilistic crossover and classical update structure, (ii) stabilizes convergence with a lock mechanism that temporarily freezes relevant variables after failed attempts, and (iii) maintains the feasibility of each individual and increases the valid solution rate by using bound, repair, and penalty structures. These features significantly improve the algorithm’s performance, convergence speed, and solution quality compared to the original ABC.

#### 5.1.1. Crossover Operator

Candidate solutions are generated through probabilistic crossover mechanisms in the employee and onlooker bee stages. In each iteration, a random neighbor k is selected for the current resource i. A crossover operation is performed, generating a new individual by exchanging information from the two parents at the genetic level, based on a predefined value p. Conversely, with probability 1-p, the traditional difference-based update of the original ABC (Equation (41)) is used. In this way, the basic operating cycle of the ABC algorithm (employee, onlooker, and scout stages) is preserved, while the method of generating new individuals is adaptively determined by a probabilistic control switch. The probabilistic switch operates at the individual update level during both the employee and onlooker phases. For each candidate generation attempt, a uniformly distributed random number r ∈ [0, 1] is generated. If r ≤ *p_cx_*, one of the predefined crossover operators is applied between the current solution and a randomly selected neighbor. Otherwise (r > *p_cx_*), the classical ABC difference-based update rule (Equation (41)) is executed. This decision is made independently for each update attempt, allowing different individuals within the same iteration to follow different search strategies. The crossover probability *p_cx_* remains fixed throughout a run, and therefore controls the expected proportion of crossover-based versus difference-based updates during the search process.

The main advantage of this design lies in its ability to dynamically adjust the exploration–exploitation balance during the search process. When the crossover operation is activated, information between solutions is transferred in a more balanced manner. In this way, new individuals are produced through less disruptive modifications, and the diversity within the solution space is maintained. When the difference-based update is applied, a broader search is performed to explore new regions; however, the capability for local refinement remains limited in this phase. By probabilistically combining these two mechanisms, the algorithm’s behavior can be dynamically adapted to the complexity of different problem landscapes.

Although the integer representation guarantees that each gene remains within predefined resource bounds, it does not by itself enforce all operational constraints arising from inter-resource dependencies. The crossover operator preserves structural feasibility at the variable level because gene exchanges occur only between valid integer-encoded individuals within these bounds. As a result, offspring remain syntactically valid in terms of resource indexing. However, operational feasibility in this problem depends on coordinated relationships among multiple resources, including time compatibility, ADR compliance, and location consistency. Such constraints cannot be fully guaranteed by representation or crossover alone. For this reason, feasibility is maintained through a hybrid structure in which integer encoding ensures bounded validity, while the repair and penalty mechanisms described in Section 4.3 enforce higher-level operational requirements.

In this study, 17 different integer-based crossover operators were tested in this phase. These operators were designed to preserve both the diversity and the structural integrity of the population. Their detailed definitions and example outputs are presented in Table 4, which summarizes how these 17 methods behave when applied to two 8-dimensional candidate solutions S_1_ =[1 2 3 2 5 2 1 3], S_2_ = [5 5 3 5 1 3 5 4]) Each operator produces one or new solutions, denoted as $C_{1}$ and $C_{2}$.

#### 5.1.2. Lock Mechanism

Probabilistic crossover has significantly improved the original ABC algorithm. However, some issues still remain in integer decision variables. Repeated unsuccessful attempts on the same dimensions lead to unnecessary evaluation costs, and the narrowing of the population in a single direction triggers premature convergence.

In this study, a lock system is added to the ABC framework to alleviate these problems. The basic idea is that if the dimension(s) modified in an iteration do not yield improvement, they are locked for a short tenure and kept fixed at the current solution’s values in subsequent candidate generations. When the tenure expires, the lock is automatically lifted and the dimension becomes searchable again.

The mechanism is operator-independent and works in both the employee and onlooker phases. First, a candidate is generated. Next, the individual’s lock vector is checked; if some dimensions are locked, the corresponding components of the candidate are masked with the current solution’s values. After evaluation, two cases arise:

- If there is improvement, the individual is updated and the failure counter is reset
- If there is no improvement, the dimensions actually modified in this iteration are identified and each receives a lock duration of LockTenure.

The lock decision rule operates at the gene level and is activated only when a candidate solution fails to improve the current individual. During each candidate-generation step, the algorithm records which dimensions have been modified. If no improvement is achieved, only those modified dimensions are assigned a positive lock duration equal to LockTenure, while unchanged dimensions remain free. The LockTenure parameter is determined based on the parameter sensitivity analysis presented in Section 6.2, where different tenure values were experimentally evaluated. This design prevents repeated unsuccessful modifications on the same dimensions while maintaining sufficient flexibility for subsequent exploration.

During each candidate generation (in both the employee and onlooker phases), all lock durations decrease by 1; once a duration reaches 0, the corresponding dimensions are released. When the scout phase renews an individual, the lock vector is also reset, preventing long freezes and preserving diversity.

From a theoretical perspective, the lock mechanism does not alter the fundamental convergence structure of the classical ABC algorithm. Since locking is temporary and each gene is automatically released after a finite number of unsuccessful update attempts, no dimension becomes permanently inaccessible. In addition, the scout phase preserves global search capability by reinitializing stagnated individuals together with their lock vectors. Therefore, the reachable solution space remains unchanged, and the probabilistic convergence characteristics of the original ABC framework are structurally maintained. The lock mechanism modifies only short-term search behavior by stabilizing unsuccessful moves, without restricting long-term exploration.

This locking mechanism offers two main benefits. First, it improves stability by temporarily fixing unsuccessful dimensions, which reduces redundant attempts and steers the search toward more promising regions. Second, it helps balance exploration and exploitation by preventing immediate repetition of ineffective moves, making local search more efficient. Once the tenure expires, the dimension is reactivated and exploration proceeds seamlessly.

In practice, the computational overhead introduced by the lock mechanism is minimal. The additional operations consist of element-wise masking, lock-vector checking, and a simple decrement update. These operations are linear with respect to the problem dimension (O(D)) for each individual and are of the same order as the standard candidate-generation step in the original ABC algorithm. Therefore, the overall time complexity per cycle remains O(NP × D), where NP denotes the population size and D represents the problem dimension. The lock mechanism increases only the constant computational factor and does not alter the asymptotic complexity of the algorithm. Consequently, it preserves the simplicity of the ABC framework while improving convergence stability and final solution quality at negligible additional computational cost.

**Figure 3:**
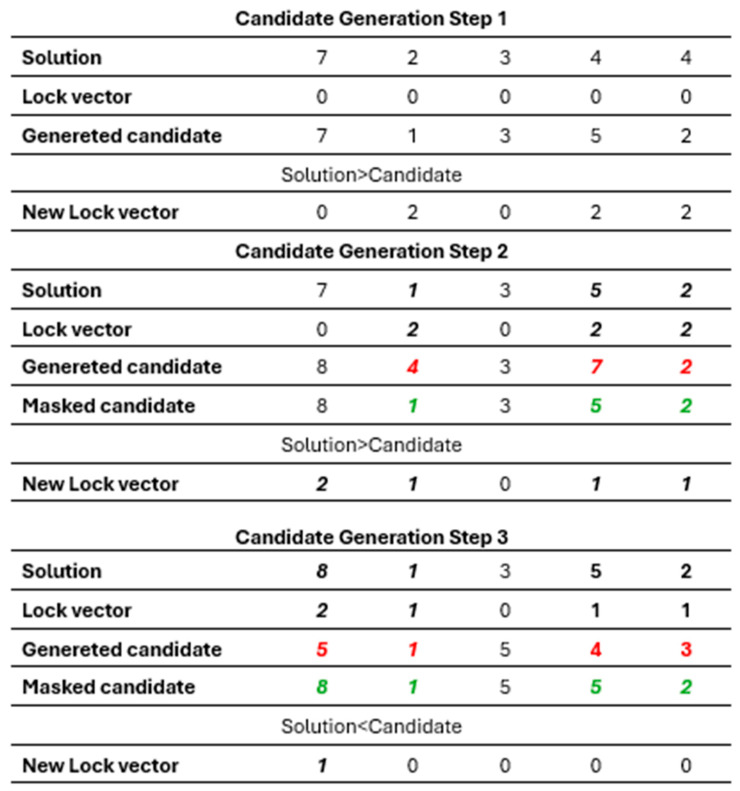
Example showing the behavior of the lock mechanism during candidate generation (red: newly generated candidate values; green: masked values reverting to the previous solution; bold: genes modified in the current iteration; italic: genes currently under lock).

To clarify how the lock mechanism behaves while generating a candidate solution, an illustrative example is provided in Figure 3. For illustration purposes, the LockTenure value in this example is set to 2 in order to clearly demonstrate how lock durations decrease over iterations and how dimensions become free again once the tenure expires. The actual LockTenure used in the experiments is determined based on the parameter analysis presented in Section 6.2.

According to Figure 3, the lock mechanism functions through three consecutive candidate-generation steps applied to the same individual. In Step 1, all lock durations are initially zero; therefore, the candidate solution is generated freely. In this attempt, the 2nd, 4th, and 5th genes are modified (e.g., 2 → 1, 4 → 5, 4 → 2). Since the candidate does not improve the current solution, lock durations are assigned only to the modified genes, resulting in the lock vector [0 2 0 2 2]. Thus, in the next candidate-generation attempt, these three genes will be masked. In Step 2, although new values are generated for the locked genes (2nd, 4th, and 5th), for example 4 for the 2nd gene, 7 for the 4th, and 2 for the 5th, these values are not accepted because the locks remain active. Instead, they are masked and replaced by the previous values of the current solution (1, 5, and 2). At the end of the process, the lock durations are decreased by one, yielding the vector [0 1 0 1 1]. Since Attempt 2 also fails to produce an improvement, additional locks are assigned to the genes that changed during this attempt. Consequently, the updated lock vector becomes [2 1 0 1 1], as shown in the table. In Step 3, a new candidate solution is generated using the lock vector [2 1 0 1 1] obtained at the end of the previous step. Although new values are produced for the 1st, 4th, and 5th genes, these genes still have positive lock durations; therefore, the newly generated values are masked and the previous solution values are retained (e.g., 5 → 8, 4 → 5, 3 → 2). At the end of Attempt 3, lock durations are decreased once more, and the lock vector becomes [1 0 0 0 0]. This indicates that only the 1st gene remains locked, while all other genes will be completely free in the next candidate-generation attempt.

### 5.2. Algorithm Flowchart and Pseudocode

The operation of the proposed method has been presented both at the algorithmic level and by visualizing it with a flowchart. The design is divided into two building blocks: Algorithm 2 includes neighbor solution generation for a single individual and the Lock mechanism; Algorithm 3 preserves the original ABC cycle (employee/onlooker/scout phases) and calls Algorithm 2 at each step.
**Algorithm 2.** Lock-Integrated Crossover/ABC-Based Neighbor Solution Generation Mechanism**Input:** i (bee index), pop, lock [i], $p_{cx}$, φ_max, bounds, LockTenure**Output:** pop [i], lock [i], counter [i]1:    **for** g = 1 to D **do** lock [i][g] ← max(0, lock [i][g] − 1)    **end for**2:    k ← RandomIndex;3:    φ ← UniformVector(−φ_max, +φ_max, D)4:  
$\;\mathrm{if}\;\mathrm{rand}\;()\;\leq \;p_{cx}$
5:               x_new ← Crossover (pop [i], pop [k])6:     **else**7:           x_new ← Equation (41)8:    **end if**9: **for** g = 1 to D **do**10:            **if** lock [i][g] > 0 **then** x_new_ [g] ← pop [i][g] **end if**11: **end for**12: x_new ← Repair (x_new, bounds)13: f_new ← Fitness (x_new)14: f_old ← Fitness (pop [i])15: **if** f_new ≤ f_old then16:            pop [i] ← x_new_17:            counter [i] ← 018: **else**19:            Jchg ← { g | x_new_ [g] ≠ pop [i][g]}20:            **for** g ∈ Jchg do lock [i][g] = LockTenure **end for**21:            counter [i] ← counter [i] + 122: **end if**

**Figure 4:**
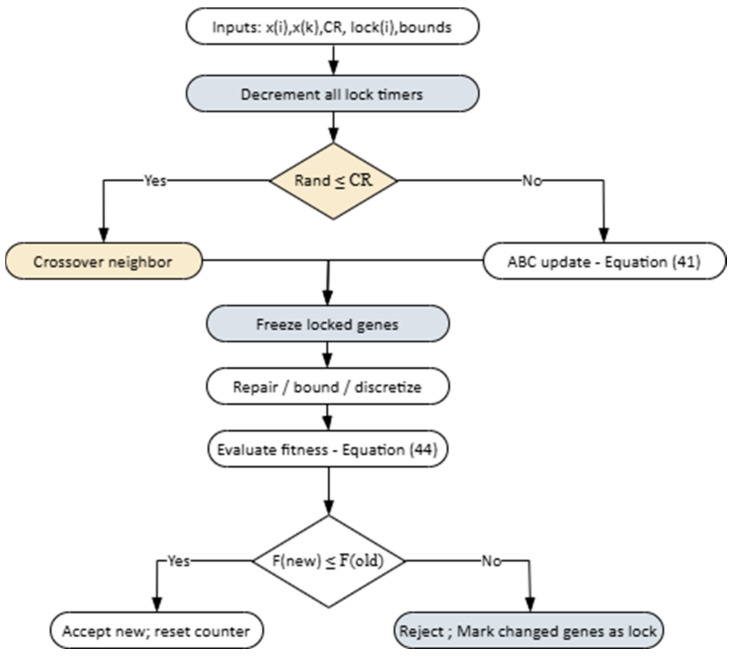
Flow Diagram of the Lock-Integrated Crossover/ABC-Based Neighbor Solution Generation Process.

The inputs of Algorithm 2 are the current individual. $x_{i}$, a randomly selected neighbor $x_{k}$, the crossover probability $p_{cx}$, and the lock vector lock(i). At the start of the loop, all lock durations are decreased by one step. Then A random number is generated and if this number is smaller than $p_{cx}$, crossover is performed, otherwise ABC-based update (Equation (41)) is performed. Next, locked genes are fixed to their previous values, the candidate solution is repaired within bounds, and the fitness is evaluated. If the new solution is better, it is accepted and the counter is reset; otherwise, lock is activated, and the lock durations of the modified genes are set to the predefined tenure value. The overall procedure is illustrated in Figure 4.

Algorithm 3 manages the entire cycle of the improved ABC. Initially, the population and Lock arrays are reset. In the employee bee phase, Algorithm 2 is called for each individual; then the fitness values are normalized, and in the onlooker bee phase, candidates are selected using the roulette wheel mechanism and Algorithm 2 is run again. If an individual fails to improve and its counter exceeds the limit, the scout phase is activated, the individual is reinitialized, and its lock vector is reset. At the end of each iteration, the global best solution is updated.
**Algorithm 3.** Main Control Structure of the Improved ABC Algorithm**Input:** PopSize, MaxIter, Limit, CR, φ_max, Tenure, bounds**Output:** x_best_, f_best_1:    Pop ← InitializePopulation(PopSize, bounds)2:    **for** i = 1 **to** PopSize **do** lock [i] ← Zeros(1, D); counter [i] ← 0 **end for**3:    (x_best, f_best) ← BestOf (Pop)4:    **for** it = 1 **to** MaxIter **do**5:            **for** i = 1 **to** PopSize **do**
6:                   (Pop [i], lock [i], counter [i]) ← Algorithm 2 (i, Pop, lock [i], CR, φ_max, bounds, Tenure, counter [i])7:            **end for**8:           P ← ComputeSelectionProbabilities (Pop)9:           // Onlooker bee phase10:            **for** m = 1 **to** PopSize_onlooker_ **do**11:                   i ← RouletteWheel (P)12:                   (Pop [i], lock [i], counter [i]) ← Algorithm 2 (i, Pop, lock [i], CR, φ_max, bounds, Tenure, counter [i])13:            **end for**14:            // Scout bee phase15:            **for** i = 1 **to** PopSize **do**16:                   **if** counter [i] ≥ Limit then17:                           Pop [i] ← RandomFeasible(bounds)18:                           lock [i] ← Zeros(1, D)19:                           counter [i] ← 020:                   **end if**21:            **end for**22:            (x_best, f_best) ← UpdateGlobalBest (Pop, x_best, f_best)23: **end for**24: **return** (x_best, f_best)

**Figure 5:**
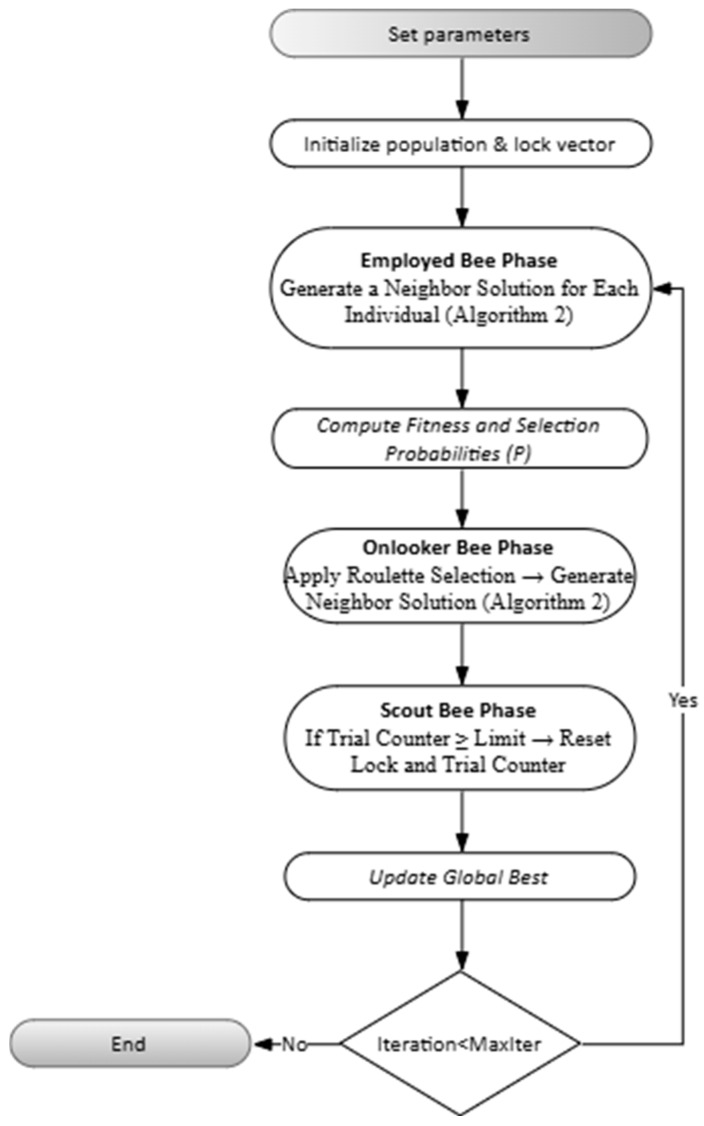
Flow Diagram of the Main Loop of the Improved ABC Algorithm.

The visual representation of the main loop presented in Algorithm 3 is provided in Figure 5. The flowchart summarizes the sequential execution of the employed bee, onlooker bee, and scout bee phases, along with the associated decision points.

## 6. Experimental Results

This section presents the complete flow and comparative evaluations of the experimental study. All algorithms were limited to 2000 iterations per run and executed on a workstation equipped with an Intel^®^ Core™ i7 processor, Intel Corporation, Santa Clara, CA, USA, (2.6 GHz) and 16 GB RAM. First, a preliminary sensitivity analysis was conducted on the small-scale dataset: the crossover probability (p_cx_) was scanned over 10 independent runs, and then multiple crossover operators were tested (10 runs each) to identify the five best candidates. Subsequently, the lock tenure parameter was tuned using another 10-run analysis. With the selected p_cx_, operator set, and lock tenure, the main experiments were performed; small-, medium-, and large-scale instances of the proposed variants (with/without lock) were compared with the baseline ABC across 30 independent runs (different random seeds). The results were summarized in tables and figures, reporting the mean, min, max, and standard deviation (std) values for each scenario. Additionally, the obtained results were compared with manually planned schedules generated by a human planner, quantitatively demonstrating the improvements of the proposed method in terms of cost, balance, and convergence performance; full compliance with operational and regulatory constraints was maintained in all experiments.

### 6.1. Data

In this study, the resource–order assignment problem in liquid freight transportation was investigated using real field data from a large-scale logistics company operating in Turkey. In each planning instance, the assignment of resources such as drivers, trucks, trailers, and ISO tanks to orders was addressed with the objectives of reducing costs and increasing operational efficiency, while feasibility was ensured through capacity, time/location compatibility, and especially ADR (dangerous goods transportation) requirements. Because ADR rules demand compliant equipment, certified drivers, and additional safety steps, the dependencies among resources increase, and the overall combinatorial structure of the problem becomes more complex. The datasets were categorized into three scales based on two criteria defined in collaboration with industry experts: (i) total number of orders and (ii) the ratio of orders requiring ADR compliance, which reflects regulatory constraint density. Sets with approximately 30–35 orders and a low ADR ratio were classified as “small,” whereas sets with a similar number of orders but a high ADR ratio were classified as “medium.” Sets containing more than 45 orders were classified as “large.” In the small dataset, 5 out of 31 orders (16%) required ADR-compliant resources, while in the medium dataset, 20 out of 34 orders (59%) were ADR-based. Since ADR orders require the simultaneous availability of certified drivers, ADR-compliant trucks, and compatible ISO tanks, the feasible assignment combinations decrease significantly as the ADR ratio increases. Therefore, problem scale in this study reflects not only workload size but also constraint intensity; consequently, even datasets with similar order counts may exhibit substantially different combinatorial complexity levels. The classification of datasets is summarized in Table 5.

The availability, ADR compliance, and total workload of the resources used in the planning are summarized in Table 6. For trailers and ISO tanks, the number of “positions” (loading–transport–unloading) is provided, while for vehicles and drivers, the total kilometers traveled in the most recent period are reported. These values are used to ensure a balanced utilization of resources.

For example, in the small-scale dataset, 112 out of 128 trailers are ADR-compliant, with a total of 4472 recorded positions; all 164 trucks possess ADR certificates and have accumulated a total of 612,058 km. For the 142 ISO tanks, 2204 positions are recorded, while out of 142 drivers, 49 hold ADR certifications and have covered a total of 592,823 km. Similar values are also present at medium and large scales.

### 6.2. Parameter and Crossover Analysis

The proposed method was quantitatively analyzed to examine the effect of the $p_{cx}$ parameter on performance. The Uniform crossover technique was used for this analysis. The $p_{cx}$ value was scanned in the range of 0.2–0.9, and for each setting, 10 independent runs were performed. The mean, min, max, and std statistics were reported. The detailed results of this parameter analysis are presented in Table 7.

The results indicate that the configuration with *p_cx_* = 0.3 achieved the lowest mean value (17.8099) while maintaining relatively stable performance (Std = 1.3666). In contrast, *p_cx_* = 0.7 exhibited higher variability (Std = 2.4441) and a weaker worst-case outcome (Max = 23.6518), suggesting potential instability when crossover is applied too frequently. Settings near the extremes (0.2 and 0.9) produced comparable but slightly higher mean values. Although *p_cx_* = 0.5 yielded the lowest standard deviation (Std = 1.2136), its mean performance remained above that of 0.3. Overall, several parameter settings can be considered statistically comparable in terms of final fitness values. Nevertheless, *p_cx_* = 0.3 consistently provided a favorable balance between mean performance, variability, and convergence behavior across runs. For this reason, it was selected as a robust reference configuration and fixed for the subsequent experiments.

Different crossover operators were compared to evaluate how effectively they balanced exploration and exploitation, improved convergence speed, and preserved diversity in discrete solutions. The statistical performance results of the 17 crossover techniques are summarized in Table 8.

The results show that the Uniform crossover achieved the best overall performance with the lowest mean value (17.81) and small variation (Std = 1.37), providing stable and effective solutions. The Inversion and Segregation operators followed with slightly higher means (35.21 and 41.90), but still showed consistent behavior, while Highly Disruptive and Ring crossovers resulted in higher mean values (57–74) and reduced stability. Among the remaining operators, those with mean values below 1000 (Discrete, Two-Point, Shuffle, Segmented, Single-Point, and Reduced Surrogate) produced weaker but still feasible results, whereas operators in the 1000–2000 range (Arithmetic and Multi-Point) showed further performance degradation. Operators with mean values above 2000 (CMX, Directional Heuristic, Flat, and Average) consistently failed to find valid or low-cost solutions, often receiving penalty values.

In conclusion, the findings clearly demonstrate that the Uniform operator outperforms others in terms of both mean performance and standard deviation. This method maintained a balanced exploration–exploitation structure during the search process and achieved significantly lower fitness values while preserving the structural consistency of the integer representation. Uniform crossover outperforms arithmetic-based operators because the problem is discrete and assignment-based. In this formulation, each gene represents a categorical resource index rather than a continuous numerical value. Arithmetic operators assume numerical continuity and generate intermediate values through averaging or linear combinations. Although a rounding procedure is applied to convert these values into valid integers, the intermediate numerical operations do not necessarily reflect meaningful structural relationships in a discrete assignment context and may still disrupt combinatorial consistency. In contrast, Uniform crossover directly exchanges gene values between parents, preserving feasible assignment structures and maintaining combinatorial building blocks. Therefore, it is better aligned with the discrete nature of the problem. Nevertheless, rather than focusing on a single operator, this study aimed to investigate alternatives with different structural characteristics. Therefore, the top five crossover methods in terms of performance and stability (Uniform, Inversion, Segregation, Highly Disruptive, and Ring) were selected for the next experimental phase. This selection ensured the inclusion of operators with both low mean fitness and acceptable standard deviation to preserve diversity.

After determining the crossover strategies, the lock duration parameter was analyzed in detail. This analysis was conducted using the best-performing Uniform operator to isolate and clearly observe the influence of LockTenure on convergence behavior. The LockTenure parameter determines how long a modified gene remains temporarily fixed after an unsuccessful update, directly affecting the balance between stabilization and continued exploration. The tested range (5–35 iterations) was selected relative to the fixed maximum number of algorithm cycles (MaxCycle). LockTenure values were defined as small fractions of the total iteration budget to prevent excessive freezing of dimensions while still allowing sufficient stabilization. Very small tenure values may release dimensions too quickly and produce negligible locking effects, whereas excessively large values may suppress exploration and lead to stagnation. For this reason, a moderate interval was systematically evaluated to achieve a balanced exploration–exploitation trade-off.

As observed in Table 9, the mean fitness values remained relatively stable within the interval of 5–25 iterations. However, beyond 25 iterations, a gradual increasing trend in the performance values was observed. In particular, the mean fitness values at tenure levels of 30 and 35 showed higher values compared to the 5–25 range, suggesting that longer locking durations may progressively restrict the search process. Therefore, the upper bound of the tested interval was limited to 35 iterations, as further increases were not expected to provide additional performance improvements. This observation supports the interpretation that LockTenure acts as a time-scale regulator in the search dynamics and should be maintained within a moderate range to preserve an effective exploration–exploitation balance.

The results show that the best performance was achieved at a Lock Tenure of 10, with the lowest mean value (14.69) and low deviation (Std = 1.06). Compared to this setting, increasing the tenure to 30 raised the mean to 17.10 and to 35 further increased it to 18.43, showing a clear deterioration of about 16% and 25%, respectively. This confirms that overly long locking durations reduce the algorithm’s efficiency by limiting exploration. In conclusion, a lock duration of 10 iterations was determined to be the most suitable parameter in terms of performance–stability balance. This configuration prevents early exploration losses seen in short locks, avoids excessive stagnation caused by long locks, and ensures a well-balanced exploration–exploitation interaction.

**Figure 6:**
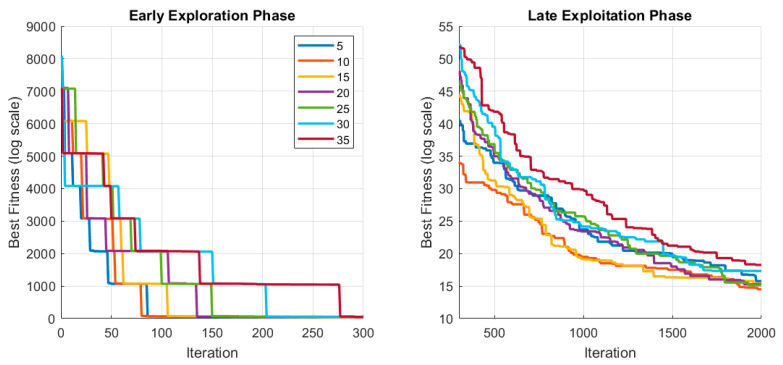
Convergence Behavior under Different LockTenure Values.

To better understand how LockTenure influences the search dynamics throughout the optimization process, convergence curves for different tenure values are illustrated in Figure 6.

As observed in Figure 6, shorter tenure values (5–15) enable a faster reduction in fitness during the early exploration phase, indicating more flexible search movements. In contrast, longer tenure values (30–35) show delayed convergence and extended plateau regions, suggesting restricted search adaptability. During the late exploitation phase, the tenure value of 10 maintains steady improvement without excessive oscillation, while larger tenure values exhibit slower refinement. These observations support the statistical findings in Table 9 and confirm that moderate locking durations provide a better balance between search flexibility and stability.

After this analysis, the final parameter settings were determined. The crossover probability was set to $p_{cx}$ = 0.3, which gave the most balanced results. The lock duration was set to 10 iterations, as this value improved stability and prevented the algorithm from converging too early. Additionally, based on the crossover comparison, five operators, namely Uniform, Inversion, Segregation, Highly Disruptive, and Ring, were identified as the best performers in terms of stability and efficiency and were selected for evaluation in the subsequent experiments.

### 6.3. Main Evaluation

The final performance evaluations of the proposed lock mechanism and the five selected crossover strategies were conducted across three different problem groups: small-, medium-, and large-scale. Each configuration was tested over 30 runs and analyzed statistically. In each experiment, the selected parameter values ($p_{cx}=0.3$, lock tenure = 10) were kept constant, and comparisons were made only among the crossover operators. Table 10 presents the effects of the proposed lock mechanism and different crossover strategies on small, medium, and large-scale problem sets. The results were evaluated based on the mean, min, max, and std values.

When small-scale problems are examined, it is observed that all operators produced better and more stable results when the lock mechanism was added. In particular, the Uniform operator achieved the best performance with an average value of 14.94 and demonstrated a highly stable performance with a low std of 1.17. In contrast, when the lock mechanism was disabled (No Lock), significant performance losses occurred across all methods; especially, the average value of originals ABC increased up to 2584. This difference indicates that the lock mechanism significantly improves convergence stability during the search process.

A similar situation is observed in medium-scale problems. When the lock mechanism was enabled, the best average value was again obtained with the Uniform operator (16.40). The average values of other methods ranged between 26 and 29. However, when the lock mechanism was not used, the performance dropped dramatically; for example, the average value of the Ring operator increased from 28.95 to 686, and that of Original ABC rose from 35.15 to 4390. These results show that, particularly in medium-scale problems, the lock mechanism maintains algorithmic stability by preventing unbalanced transitions in the solution space.

In large-scale problems, the cost values naturally increased across all methods. However, the lock mechanism still provided a significant advantage. With the lock enabled, the Uniform and Inversion operators produced the best results with average values of 26.77 and 46.33, respectively. In contrast, when the lock mechanism was disabled, the average of the Ring operator increased from 55.41 to 2420, and that of Original ABC rose from 58.30 to 6990. The increase in std values also indicates that convergence became unstable in this case.

Overall, across all three problem scales, the Uniform operator achieved the best results with both the lowest mean and the lowest std values. It can be said that this operator maintains diversity in the solution space while balancing its exploitation ability. The lock mechanism, on the other hand, increased algorithmic stability by preventing solution degradation and oscillatory search behavior, especially in large-scale problems. To clearly demonstrate the numerical impact of enabling the lock mechanism, the mean cost values obtained with the lock mechanism were compared with the mean cost values produced without it, and the percentage differences between the two were calculated. The resulting improvement rates (Improvement %) are presented in Table 11.

The improvement calculation is given by the following equation:

(45) $$Improvement\left(\%\right)=100\frac{NoLock_{Mean}-Lock_{Mean}}{NoLock_{Mean}}$$

When examining the results in Table 11, it is clear that the lock mechanism improves performance at all problem sizes. In small-scale problems, improvement rates range between 16% and 49% for most operators, while Ring (66.6%) and Original ABC (98.8%) show much higher gains. In medium-scale problems, the same pattern continues, with moderate improvements for most operators (12–46%) and significant gains for Ring (95.8%) and Original ABC (99.2%). In large-scale problems, the lock mechanism remains highly effective, achieving over 90% improvement for both Ring and Original ABC, while other operators such as Uniform (71.2%) and Inversion (62.4%) also show consistent benefits. These results confirm that the lock mechanism significantly enhances performance and maintains its effectiveness even as problem complexity increases.

**Figure 7:**
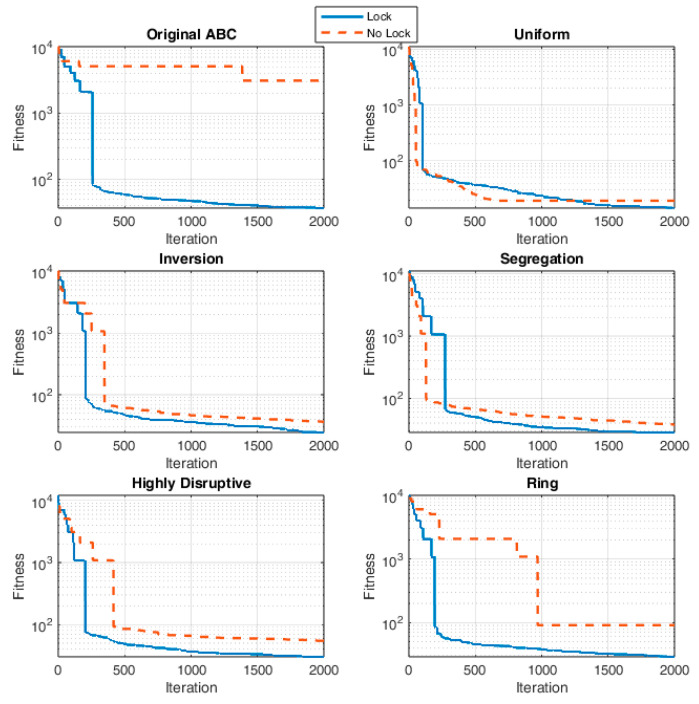
Convergence Behavior of Lock vs. No-Lock Configurations for Different Crossover Operators.

Lock and No-Lock configurations for each crossover operator are presented in Figure 7.

This figure illustrates the effect of the lock mechanism on the convergence behavior of the algorithm for a medium-scale problem. In all operators, the Lock configuration provided a more stable improvement throughout the iterations. Particularly in the Ring and Original ABC operators, it was observed that when the lock mechanism was disabled, the solution cost remained at high levels, whereas when the lock was enabled, convergence occurred both earlier and more stably. These results demonstrate that the lock structure strengthens the overall algorithm performance by reducing fluctuations during the search process. In the Uniform operator, when the Lock system was not applied (NoLock), it initially showed rapid convergence but soon stagnated; in contrast, with the Lock system enabled, a more balanced and stable decrease was observed, resulting in lower cost values at the end of the process.

To evaluate the effect of the lock mechanism on multiple objectives, the results of eight different objective functions are presented in Table 12.

When Table 12 is examined, it is observed that better results were obtained in cases where the Lock system was applied. The success of the lock mechanism is particularly evident in the Original ABC operator, where the Z_2_ value decreased from 22.71 to 10.13, the Z_4_ value from 15.50 to 5.80, and the Z_7_ value from 24.07 to 5.60. In the Ring operator, the Z_2_ objective decreased from 17.91 to 7.28, and the Z_6_ objective from 21.71 to 9.34. Additionally, for the Z_7_ and Z_8_ objectives, the Lock configuration produced much lower costs compared to the No-Lock version. In the Segregation operator, lock success is seen in Z_4_ (5.01 → 3.58) and Z_6_ (17.57 → 11.97). The Uniform and Inversion operators show more balanced results. In the Uniform operator, the No-Lock system produced slightly lower values than the Lock configuration; for example, in the Z_2_ objective, 3.44 (No-Lock) is slightly better compared to 4.38 (Lock). However, in Z_3_ (2.32 → 1.33), Z_6_ (7.34 → 5.81), and Z_7_ (2.40 → 1.37), the Lock configuration exhibited superior performance. Overall, it can be concluded that the lock system provided significant improvements in objectives focused on Z_2_, Z_4_, and Z_6_–Z_7_ for almost all operators, while demonstrating limited but stable performance in other objectives.

In order to provide a broader performance assessment, the proposed Lock-Uniform configuration was compared with several well-known metaheuristic algorithms, including Genetic Algorithm (GA), Particle Swarm Optimization (PSO), Tunicate Swarm Algorithm (TSA), Seagull Optimization Algorithm (SOA), and Ant Colony Optimization (ACO). All algorithms were implemented under the same iteration limit and experimental conditions. Each configuration was executed over 30 independent runs, and statistical performance measures (mean, min, max, and standard deviation) were recorded for small, medium, and large-scale instances. The comparative results are presented in Table 13.

The results indicate that the Lock-Uniform configuration consistently achieved the lowest mean cost values across all problem sizes. GA produced competitive but consistently weaker results compared to the proposed approach across all problem sizes, with the performance gap becoming more pronounced as the problem scale increased. Swarm-based algorithms originally designed for continuous optimization, such as PSO, TSA, and SOA, exhibited substantially higher mean values and large variance. It is important to note that fitness values exceeding 1000 correspond to solutions that violate feasibility constraints and therefore incur penalty costs. The high mean and maximum values observed for these algorithms indicate that a considerable portion of their runs failed to reach the feasible region within the iteration limit. Although some runs produced feasible solutions, the algorithms were not consistently able to escape the penalty-dominated region. This behavior suggests that continuous swarm dynamics, even when combined with integer adaptation, may struggle to efficiently navigate highly constrained discrete assignment landscapes. ACO demonstrated more stable behavior than other swarm algorithms but remained inferior to the proposed approach in terms of mean performance.

The manual planning results correspond to the actual operational plans generated by the dispatch planners of a leading logistics company in Turkey. The data represent live operational assignments carried out in daily practice. Planning is performed under real-time operational pressure, where multiple incoming orders are typically handled sequentially rather than within a unified optimization framework.

In practice, dispatchers assign drivers, trucks, trailers, and ISO tanks to orders based on experience, feasibility checks, and operational urgency. Due to the combinatorial complexity of simultaneously matching multiple resource types under ADR constraints, orders are often planned independently rather than jointly optimized. While this approach ensures feasibility and operational continuity, it does not systematically minimize global cost imbalance, workload deviation, or multi-objective inefficiencies across the entire order set. Therefore, the comparison presented below reflects the structural difference between sequential human planning and simultaneous combinatorial optimization.

Lock_Uniform_ approach, obtained by applying the lock system to the best-performing Uniform operator, was compared with solutions produced by manual planning. The results are presented in Table 14.

According to Table 14, the Lock-Uniform approach demonstrates effective performance across different problem scales. It has shown a clear superiority over manual planning, particularly in the Z_2_, Z_4_, Z_6_, and Z_7_ objectives. For instance, in small-scale problems, the Z_2_ value decreased from 19.73 to 4.46, and the Z_4_ value dropped from 16.75 to 0.95. Similarly, in medium-scale problems, the Z_6_ value declined from 21.40 to 5.81, while in large-scale problems, the Z_7_ value decreased from 32.39 to 3.91. These results indicate that the Lock-Uniform approach maintains solution quality as the problem size increases and produces more balanced and lower-cost solutions compared to manual planning.

## 7. Conclusions and Future Work

This study proposes an enhanced Artificial Bee Colony framework that integrates a probabilistic Uniform crossover operator and a gene-level lock mechanism for discrete and highly constrained multi-resource allocation problems. The experimental findings demonstrate that the two mechanisms provide complementary benefits.

When applied independently on small-scale instances, the crossover-enhanced ABC reduced the mean cost of the Original ABC from 2584.01 to 17.79, while the lock-only configuration reduced it to 29.78. It should be noted that the extremely high mean value of the Original ABC is mainly due to penalty-dominated solutions (fitness values exceeding 1000), indicating that many runs failed to reach the feasible region within the iteration limit. When both mechanisms were combined, the mean cost further decreased to 14.94, confirming that controlled gene exchange and temporary stabilization jointly improve convergence reliability.

Across small-, medium-, and large-scale datasets, the Lock–Uniform configuration consistently achieved the lowest mean and standard deviation values. Improvement rates exceeded 90% compared to the No-Lock Original ABC and remained substantial even in large-scale instances. The lock mechanism significantly reduced oscillatory behavior and prevented repeated unsuccessful updates, particularly under high ADR constraint intensity.

In the broader comparative evaluation, the proposed method outperformed established metaheuristic algorithms, including GA, PSO, TSA, SOA, and ACO, under identical experimental conditions. While GA produced feasible and competitive solutions, swarm-based algorithms originally designed for continuous domains frequently remained in penalty-dominated regions and exhibited high variance. These findings indicate that discrete structural preservation and controlled stabilization are critical in highly constrained assignment problems.

Finally, comparisons with real operational plans generated by professional dispatchers showed consistent improvements in workload balance and cost-related objectives. The results confirm that simultaneous integrated optimization provides measurable advantages over sequential manual planning under complex regulatory and operational constraints.

Future research may investigate adaptive control of the crossover probability. Although a fixed value (p_cx_ = 0.3) provided stable and competitive performance in this study, dynamically adjusting p_cx_ during iterations may further improve convergence stability and search efficiency, particularly in large-scale or highly constrained instances. In addition, while the present study employs a weighted-sum formulation to obtain a single implementable solution, extending the framework to true multi-objective optimization approaches could provide deeper insight into trade-offs among conflicting objectives. Exploring Pareto-based ABC variants or hybrid multi-objective metaheuristics represents a promising direction for enhancing decision-support flexibility in complex logistics planning environments.

## Figures and Tables

**Table 1:** Detailed Overview of Resource Information.

Driver	ISO tank	Trailer	Tractor
Departman	Departman	Departman	Departman
ID	ID	ID	ID
Full Name	Tank Number	License Plate	License Plate
Status	Type	Trailer Type	Tractor Type
Date of Birth	Tank Capacity	Payload Capacity	ADR Status
Driver’s License	Product Group	Ownership Status	Brand
Driver’s License Expiry Date	Number of positions visited in the last 1 month	Product Group	Model
SRC 5 License		ADR Status	Fuel Efficiency
		Total distance (km)	Total distance (km)
			Product Group

**Table 3:** Example conflict matrix showing time overlaps between orders.

Orders	1	2	3
1	1	0	1
2	0	1	0
3	1	0	1

**Table 4:** Definitions and Example Outputs of the Eighteen Integer-Based Crossover (Recombination) Operators Used in This Study.

No	Method	Description	C_1_	C_2_
1	Uniform	Each gene is taken from one of the parents with a certain probability	[1 5 3 2 5 3 1 3]	[5 2 3 5 1 2 5 4]
2	Inversion	A segment of S2 is reversed and transferred to S1	[1 2 3 1 5 3 1 3]	—
3	Segregation	A single segment is mutually exchanged between solutions	[1 2 5 3 5 1 1 3]	—
4	Highly Disruptive	Some variables of a solution are randomly replaced with those from another solution.	[1 5 3 2 4 2 1 3]	[2 5 3 5 1 3 5 5]
5	Ring	The decision variable of S2 is circularly shifted	[1 1 3 5 5 5 1 3]	[5 5 3 1 1 1 5 3]
6	Discrete	Each decision variable is taken randomly from one parent	[1 5 3 2 1 2 1 4]	—
7	Two-Point	Segment exchange between two crossover points	[1 2 3 5 1 3 1 3]	[5 5 3 2 5 2 5 4]
8	Shuffle	Genes in both solutions are initialized identically, a single-point crossover is performed, and they are restored to the new, previously shuffled solution.	[1 5 3 5 5 3 1 4]	[5 2 3 2 5 2 1 3]
9	Segmented Integer	Solution divided into segments and swapped in sequence	[1 2 3 5 5 2 5 4]	[5 5 3 2 1 3 1 3]
10	Single-Point	Single cut point used for segment swap	[1 2 3 2 5 3 5 4]	[5 5 3 5 1 2 1 3]
11	Reduced Surrogate	Positions where the solutions have different variable values are identified, one of these positions is randomly selected, and a single-point crossover is performed at that point.	[1 2 3 2 1 3 5 4]	[5 5 3 5 5 2 1 3]
12	Arithmetic (α = 0.4)	Each variable of the offspring is generated as a weighted average of the corresponding variables of the two parent solutions using a random coefficient α	[3 3 3 3 3 2 3 3]	[3 4 3 4 3 3 3 4]
13	Multi-Point	Multiple crossover points used	[1 5 3 5 1 3 5 4]	[5 2 3 2 5 2 1 3]
14	CMX	A new solution is generated by combining the arithmetic mean and the product of two parent solutions, representing both their average and multiplicative relationship.	[2 3 3 3 2 2 2 3]	[3 4 3 4 3 3 3 4]
15	Directional Heuristic	Adds weighting to the difference vector	[5 6 3 7 3 3 9 4]	—
16	Flat	Each variable of the new solution is randomly selected between the corresponding values of two parent solutions, producing an offspring that lies within their range.	[2 4 3 3 4 2 5 3]	—
17	Average	Average of each gene position	[3 4 3 4 3 3 3 4]	—

**Table 5:** Order Summary.

Category	Total Orders	ADR Orders
Small	31	5
Medium	34	20
Large	49	19

**Table 6:** Resource Information.

Category	Resource	Count	ADR Compliant	Total Positions	Total Distance (km)
Small	Trailer	128	112	4.472	—
	Truck	164	164	—	612.058
	ISO tank	142	142	2.204	—
	Driver	142	49	—	592.823
Medium	Trailer	141	120	4.380	—
	Truck	177	177	—	625.200
	ISO tank	152	152	2.176	—
	Driver	152	64	—	606.148
Large	Trailer	148	124	4.882	—
	Truck	181	181	—	708.144
	ISO tank	157	157	2.391	—
	Driver	159	69	—	700.568

**Table 7:** The effect of p_cx_ value on optimization performance.

p_cx_	Mean	Min	Max	Std
0.2	18.5585	16.0907	20.5851	1.4569
0.3	17.8099	15.2790	20.0126	1.3666
0.4	19.0635	16.7554	21.8896	1.4943
0.5	18.4329	17.0714	20.4192	1.2136
0.6	18.2258	15.8769	20.5814	1.5318
0.7	18.2241	14.4805	23.6518	2.4441
0.8	17.9867	15.2656	20.0947	1.4781
0.9	18.4958	15.5101	21.0169	1.5110

**Table 8:** Statistical performance comparison of integer-based crossover techniques used in the ABC algorithm.

Crossover Technique	Mean	Min	Max	Std
Uniform	17.8099	15.2790	20.0126	1.3666
Inversion	35.2130	32.7394	38.9895	2.0790
Segregation	41.9059	34.3040	44.9741	3.0670
Highly Disruptive	57.6185	52.7558	64.2296	3.0369
Ring	73.7986	69.1655	86.8266	4.9663
Discrete	223.2071	21.2514	1023.5356	399.5710
Two-Point	332.2750	26.7472	1036.3769	458.9136
Shuffle	428.1476	25.3161	1031.3183	489.8360
Segmented	478.5645	63.9312	1089.7815	490.8220
Single-Point	847.2512	43.4063	2047.4486	600.5156
Reduced Surrogate	849.2719	51.6198	2049.9507	598.3603
Arithmetic	1264.0993	56.2436	2074.2065	873.1853
Multi-Point	1560.1160	64.2015	3066.9344	806.2371
CMX	2886.2910	2094.1157	3087.9357	394.7244
Directional Heuristic	3082.8826	2100.3122	4076.0948	441.8592
Flat	3083.2549	2086.8505	4079.0674	445.4837
Average	3085.0669	3074.2968	3100.2184	7.3326

**Table 9:** The impact of lock tenure on optimization performance.

Lock Tenure	Mean	Min	Max	Std
5	15.5778	12.8540	18.1776	1.3958
10	14.6902	13.0543	16.3664	1.0612
15	15.0020	12.8102	16.5706	1.1348
20	15.2705	13.2617	18.3100	1.2119
25	15.0380	13.8208	16.3655	0.8064
30	17.1004	16.0430	18.1376	0.7095
35	18.4296	16.5944	19.3498	0.7813

**Table 10:** Comparative performance of the lock mechanism and crossover strategies across different problem sizes (small, medium, and large).

			Uniform	Inversion	Segregation	Highly Disruptive	Ring	Original ABC
Small	Lock	Mean	14.9455	23.0970	23.7918	25.0048	24.5426	29.7852
		Min	11.6528	19.6538	20.3487	21.5028	20.1540	24.3433
		Max	17.3520	25.1803	26.0937	27.1693	27.7086	32.2309
		Std	1.1713	1.1999	1.2944	1.5365	1.6437	1.7820
	No Lock	Mean	17.7881	31.3269	34.1555	48.9672	73.4584	2584.0156
		Min	14.2552	24.4396	26.4725	38.3175	64.8503	1088.9215
		Max	20.5825	38.9895	44.9741	64.2296	86.8266	3101.2159
		Std	1.6793	3.4963	6.1614	6.8901	3.9096	616.2699
Medium	Lock	Mean	16.4000	26.5704	27.2468	28.9392	28.9484	35.1571
		Min	14.1110	24.2978	23.7767	23.9971	25.0086	31.0225
		Max	18.3959	31.7632	30.0342	32.1627	31.4554	38.7595
		Std	1.1818	1.8131	1.7792	1.6057	1.5207	1.5240
	No Lock	Mean	18.7708	37.3283	35.8263	54.0699	686.8777	4390.738
		Min	15.8906	31.0365	30.5856	48.6715	75.6377	3091.815
		Max	21.2774	43.5979	41.2310	58.9083	2084.5919	5103.104
		Std	1.3190	3.0977	2.2891	2.4197	609.5651	639.2047
Large	Lock	Mean	26.7695	46.3267	47.3517	51.8959	55.4114	58.3063
		Min	23.0721	41.1261	41.9151	46.4426	48.5026	52.0950
		Max	30.8887	50.0496	50.7725	55.5375	59.7567	63.5160
		Std	1.9538	2.0486	2.1149	1.8917	2.3255	2.3381
	No Lock	Mean	92.9376	123.3524	56.7507	84.1060	2420.2641	6990.4017
		Min	22.1619	48.4650	47.8486	77.4886	1108.7986	5124.2675
		Max	1026.9315	1067.2099	70.6730	93.9027	4114.0513	8123.0123
		Std	249.2000	251.3051	4.2014	4.1484	736.5223	668.8604

**Table 11:** Improvement (%) between Lock and No-Lock configurations across different problem sizes.

	Small	Medium	Large
Uniform	15.9803	12.6303	71.1963
Inversion	26.2710	28.8197	62.4436
Segregation	30.3427	23.9475	16.5619
Highly Disruptive	48.9356	46.4782	38.2970
Ring	66.5898	95.7855	97.7105
Original ABC	98.8473	99.1993	99.1659

**Table 12:** Performance Metrics of Algorithms Based on Multiple Objectives.

		Z_1_	Z_2_	Z_3_	Z_4_	Z_5_	Z_6_	Z_7_	Z_8_
Original ABC	Lock	0.5077	10.1319	2.7890	5.8023	0.0714	11.1206	5.6029	0.3636
	NoLock	0.5079	22.7091	5.1619	15.5008	0.5749	22.6304	24.0658	2.0000
Uniform	Lock	0.5005	4.3829	1.3307	0.8266	0.0000	5.8108	1.3678	0.0000
	NoLock	0.5170	3.4421	2.3169	1.0081	0.0000	7.3441	2.4002	0.0000
Inversion	Lock	0.5211	6.1397	1.7782	4.8185	0.0000	8.7109	2.4765	0.0000
	NoLock	0.5097	4.9844	2.5037	6.2334	0.0000	15.7748	5.8119	0.5000
Segregation	Lock	0.4933	6.8334	1.8656	3.5818	0.0000	11.9734	3.4441	0.0714
	NoLock	0.4819	6.2722	2.3287	5.0146	0.2500	17.5660	5.3856	0.0000
Highly Disruptive	Lock	0.5182	10.0268	2.8292	5.6840	0.0000	8.6785	2.3824	0.0000
	NoLock	0.4894	13.3793	3.9900	8.2739	0.1364	14.9689	6.8621	0.5714
Ring	Lock	0.4809	7.2780	2.2446	5.3063	0.2253	9.3360	3.9060	0.0000
	NoLock	0.4822	17.9053	4.3290	10.7229	3.5000	21.7093	16.1442	1.7500

**Table 13:** Comparative Performance Results of Lock_Uniform_, GA, PSO, TSA, SOA, and ACO Across Different Dataset Sizes.

		ABC (Lock_Uniform_)	GA	PSO	TSA	SOA	ACO
Small	Mean	14.94	18.93	1794.89	2118.60	2044.77	43.72
	Min	11.65	14.17	43.33	1079.28	1063.08	36.82
	Max	17.35	23.74	3077.13	3096.57	3082.17	52.67
	Std	1.17	2.3	774.94	604.21	793.55	3.16
	Rank	1	2	4	6	5	3
Medium	Mean	16.4	25.92	1372.50	4367.17	2918.35	59.12
	Min	14.11	21.29	66.87	3082.94	1080.24	51.26
	Max	18.40	32.68	3082.10	6093.58	5089.26	66.19
	Std	1.1818	2.83	1037.95	1011.63	1185.16	3.57
	Rank	1	2	4	6	5	3
Large	Mean	26.77	55.01	2938.07	6691.76	5245.725	92.64
	Min	23.07	46.89	1088.94	5117.217	3106.275	78.72
	Max	30.89	63.4	6113.59	9118.27	9097.343	106.53
	Std	1.95	3.79	1320.55	1332.482	1362.039	4.81
	Rank	1	2	4	6	5	3

**Table 14:** Comparison of Lock_Uniform_ and manual planning for different problem sizes.

		Z_1_	Z_2_	Z_3_	Z_4_	Z_5_	Z_6_	Z_7_	Z_8_
Small	ABC (Lock_Uniform_)	0.5111	4.4574	2.0436	0.9483	0.0000	5.0672	1.5227	0.0000
	Manuel	0.5148	19.7347	4.7109	16.7504	0.1111	24.0238	19.7524	0.0000
Medium	ABC (Lock_Uniform_)	0.5005	4.3829	1.3307	0.8266	0.0000	5.8108	1.3678	0.0000
	Manuel	0.5077	17.3270	4.9658	24.1349	0.0000	21.3992	34.8255	0.5000
Large	ABC (Lock_Uniform_)	0.5189	8.4989	2.5675	2.3524	0.0000	8.8464	3.9061	0.0000
	Manuel	0.5229	23.2843	7.8187	22.0436	0.1369	28.0845	32.3883	0.8895

## Data Availability

The data used in this study were obtained from a large-scale logistics company and are not publicly available due to confidentiality agreements. Data may be available from the corresponding author upon reasonable request, subject to permission from the data provider.

## Nomenclature

n	Number of orders
$m_{d}$	Number of drivers
$m_{c}$	Number of trucks
$m_{t}$	Number of trailers
$m_{i}$	Number of ISO tanks
$d_{i}$	Distance of order i
$y_{i}$	Fuel consumption of truck-i
$y_{max}$	Maximum fuel consumption rate (among trucks)
$D_{tot}$	Total distance of all orders
δ(j)	Driver assigned to order j
τ(j)	Truck assigned to order j
θ(j)	Trailer assigned to order j
κ(j)	ISO tank assigned to order j
$X_{j}^{city}$	Indicator variable, 1 if order j is intra-city, 0 otherwise
$X_{j}^{sort}$	Indicator variable, 1 if order j is short-distance, 0 otherwise
$K_{u}^{past}$	Distance traveled by driver u in the past month
$K_{v}^{past}$	Distance traveled by truck v in the past month
$P_{t}^{past}$	Number of positions handled by trailer t in the past month
$Q_{k}^{past}$	Number of positions handled by ISO tank k in the past month
$P_{t}^{past,city}$	Number of intra-city positions handled by trailer t in the past month
$Q_{k}^{past,city}$	Number of intra-city positions handled by ISO tank k in the past month
$S_{u}^{past}$	Number of short-distance trips made by driver u in the past
$L_{u}^{past}$	Number of long-distance trips made by driver u in the past
φ	Target intra-city ratio (e.g., φ = 0.40)
$z_{k}$	Objective function value k
$x_{i,j,v,k,t,s}\in \{0,1\}$	Assignment variable: equals 1 if order i is assigned to driver j, truck v, ISO tank k, and trailer t during time period s, and 0 otherwise.
